# Accelerated biological aging: the role of chronic inflammation, weathering and novel therapeutic strategies

**DOI:** 10.3389/fragi.2026.1863781

**Published:** 2026-07-21

**Authors:** Frances M. Marin-Maldonado, Mitzy J. Santiago-Rosario, Wilfred Fonseca-Ferrer, Cornelis P. Vlaar, Magaly Martinez-Ferrer, Jose Colon-Saez, Jorge Duconge-Soler

**Affiliations:** 1 Pharmacogenomics (PGx) Laboratory, University of Puerto Rico, Medical Sciences Campus, San Juan, PR, United States; 2 Department of Pharmaceutical Sciences, School of Pharmacy, University of Puerto Rico, Medical Sciences Campus, San Juan, PR, United States; 3 Molecular Sciences Research Center, University of Puerto Rico, San Juan, PR, United States; 4 Doctor of Pharmacy Program, School of Pharmacy, University of Puerto Rico, Medical Sciences Campus, San Juan, PR, United States; 5 Division of Cancer Clinical and Translational Research, University of Puerto Rico Comprehensive Cancer Center, San Juan, PR, United States

**Keywords:** cancer, Caribbean Hispanics, genetic biomarkers, inflammation, neurodegeneration, non-coding RNAs, precision medicine, senolytic agents

## Abstract

Biological aging is a gradual, multisystem process characterized by progressive functional decline, diminished physiological resilience, and increased susceptibility to chronic diseases. Growing evidence suggests that persistent exposure to adverse social environments, chronic psychological stress, and socioeconomic disadvantage can accelerate biological aging by altering age-related biomarkers and stress-response pathways. The weathering hypothesis provides a framework to explain how the cumulative burden of psychosocial adversity becomes biologically embedded, resulting in physiological wear and tear that hastens age-related deterioration over time. A central mechanism underlying this process is chronic low-grade inflammation, which contributes to the pathogenesis of numerous age-related conditions, including cancer and neurodegenerative diseases (NDDs). These disorders are highly prevalent among Caribbean Hispanics, a population disproportionately affected by social disadvantages and rapid population aging. Recent advances in the biology of inflammation and cellular senescence have generated interest in therapeutic strategies aimed at modulating fundamental mechanisms of aging. Emerging approaches, including senolytics, anti-inflammatory agents, and non-coding RNA–based interventions, offer promising opportunities for precision medicine; however, RNA-based therapies remain largely experimental, and their clinical translation to address stress-related aging and health disparities has yet to be established. In this review, we examine the interplay between weathering and inflammation in shaping biological aging and discuss the potential of innovative precision medicine approaches to mitigate age-related decline and reduce health disparities in vulnerable populations.

## Introduction

In Western countries, the average population age is steadily increasing, giving rise to significant challenges for healthcare systems. Specifically, by 2050, it is projected that more than 25% of the Caribbean Hispanic population will consist of older adults aged 60 years and above. One of the main factors that has led to population aging in this region is a demographic transition from elevated levels of fertility and low mortality to low levels of fertility and high mortality. This trend started in the mid-1960s and continues until today, thereby accelerating demographic aging. Exacerbating the effects of demographic aging, biological aging is regarded as a progressive, generalized impairment of function accompanied by an increased risk of vulnerability to environmental challenges and disease risk. It is well documented that socioeconomically disadvantaged individuals are at an increased risk of age-related diseases. This has resulted in the development of the “weathering hypothesisl,” which states that chronic exposure to social adversity and stress accelerates the process of aging. In this review, we want to explore biomarkers associated with weathering and present evidence linking psychosocial stress to molecular aging derived from preclinical and cross-sectional human studies. More broadly, we discuss the impact of chronic inflammation and weathering on accelerated biological aging and explore the potential for novel therapeutic strategies. We place particular emphasis on vulnerable populations such as Caribbean Hispanics, who bear a disproportionate burden of social disadvantage and rapid population aging, contributing to the high prevalence of age-related disorders in this population. The purpose of this review is to synthesize the current state of knowledge on emerging anti-aging and longevity-oriented strategies while examining their potential relevance to the Caribbean Hispanic population. In addition to demographic aging, Caribbean Hispanics experience a disproportionate burden of age-associated chronic conditions, including neurodegenerative diseases and cancer, underscoring the need to better understand the biological and social drivers that contribute to accelerated aging in this population. Despite these vulnerabilities, Caribbean Hispanics remain markedly underrepresented in clinical, genomic, and translational aging research. A critical limitation encountered throughout this review is the scarcity of population-specific data from Caribbean Hispanic cohorts. Most mechanistic evidence related to pathways such as non-coding RNAs, senolytic therapies, and longevity-associated regulators has been generated primarily in preclinical models or in Europeans. Similarly, there is a notable absence of population-based biomarker studies, epigenetic aging clock analyses, and inflammatory profiling data from Puerto Rico, the Dominican Republic, and Cuba. This lack of representation restricts direct translational interpretation and highlights an important gap in the current scientific literature.

Accordingly, this review adopts a critical perspective that distinguishes preclinical findings from clinically validated evidence and discusses areas in which results remain inconsistent or contradictory. While several longevity biotechnology approaches show promising therapeutic potential, their translational feasibility within Caribbean Hispanic populations remains uncertain due to limited regional data, healthcare infrastructure considerations, and unresolved population-specific biological and environmental factors. Rather than extrapolating conclusions unsupported by regional evidence, this work aims to place emerging anti-aging strategies within a broader global framework while identifying key scientific and translational gaps that must be addressed. By highlighting both the promise of novel longevity interventions and the absence of Caribbean-specific research, this review seeks to encourage future population-focused studies that may ultimately inform equitable implementation of aging-related therapeutics in a historically understudied region with significant potential to benefit from strategies that mitigate age-associated effects.

## Methods

The methodology for this literature review involved a systematic search of PubMed, ACS Publications, and Google Scholar between 1992–2026. The search prioritized publications from the last 10 years to ensure the inclusion of recent findings. However, older articles were included when they represented foundational studies, introduced key concepts, or provided essential background information relevant to accelerated biological aging, inflammaging, cellular senescence, weathering, or senotherapeutic strategies. The search terms included combinations of the following keywords: aging, accelerated biological aging, weathering, inflammaging, chronic inflammation, cellular senescence, senescence-associated secretory phenotype, SASP, senolytics, senomorphics, natural products, synthetic senolytics, cancer, neurodegeneration, Alzheimer’s disease, and Caribbean Hispanic populations. Keywords were used individually and in combination with up to two additional terms using the Boolean operators “AND” and “OR,” according to the requirements of each database.

Articles were selected based on their relevance, methodological rigor, and contribution to understanding the relationship between aging, chronic inflammation, and the therapeutic potential of natural and synthetic senolytic agents. A second screening of these articles was performed by applying the following criteria: (i) Full article was accessible, (ii) published in the English language, except statistical data from Cuba and Puerto Rico that were in Spanish and (iii) were not letters to editors or manuscripts. Statistical articles were focused on the Caribbean Hispanic population, specifically in Puerto Rico, the Dominican Republic, and Cuba.

The temporal distribution of the selected references showed that most articles included in this review were published within the last 5 years, with the highest concentration between 2021 and 2025. The highest number of publications was observed in 2021, with 21 articles, followed by 2023 with 18 articles, 2022 with 16 articles, and 2025 with 13 articles, as shown in Supplementary Figure S1. This pattern suggests a growing scientific interest in accelerated biological aging, inflammaging, and senotherapeutic strategies. Although the search prioritized recent literature from the last 10 years, selected older articles were included because they represented foundational studies or provided essential background information relevant to the scope of this review.

The literature reviewed in this article is predominantly preclinical, reflecting a recognized gap in robust clinical and longitudinal human studies within this emerging field. This difference stems from the novelty of senolytic and RNA-based therapeutic strategies, many of which remain in initial stages of development. While the AMSTAR tool could be used to assess methodological quality, the limited number of available studies on these topics represents an important challenge. Applying the full AMSTAR criteria may further reduce the number of eligible publications, potentially limiting the review’s comprehensiveness. Given the scarcity of evidence in this field, we opted for a broader inclusion strategy while acknowledging the methodological limitations of the available literature.

In this review, the term dementia is used to align with the terminology adopted in the original literature; however, our primary focus is on Alzheimer’s disease. It is important to acknowledge that the term dementia is increasingly being replaced in contemporary research and clinical practice to reflect a more accurate and sensitive understanding of neurocognitive disorders. Additionally, to align with the terminology used in this review, we refer to the fertility data from Cuba as the “total fertility rate”; however, the original source used the term “tasa global de fecundidad” (Spanish for “total fertility rate”). The information gathered was then organized and critically analyzed to identify molecular mechanisms, biomarkers, and clinical perspectives related to the use of these compounds in age-related diseases.

### How is population aging affecting the Caribbean Hispanic region?

Demographic aging is measured with different parameters: (1) *C(t)*: the proportion of older adults above 60, (2) *A(t)*: the mean age of the population, and (3) *L(t)*: an indicator of the availability of support among the younger generations. The first indicator, *C(t)*, is a conventional measure, and *A(t)* helps explain the growth pattern in older adults. The third one, *L(t)*, is the ratio of the adult to the elderly; it is a clue marker of available family resources or constraints in the tendency toward elderly residential arrangements (Palloni et al., 2002). Overall, these parameters help to describe the demographic nature of a growing elderly population.

The fertility rate is another parameter that helps to understand how a population is increasing or decreasing. The fertility rate is a metric used to evaluate the average number of children that women give birth to in a particular country (World Population Review, 2025). On the other hand, the total fertility rate (TFR) is a metric that essentially represents the average number of offspring that a woman is expected to have in a given country throughout her lifetime. The TFR is an important parameter that provides information on the fertility rate needed to maintain a society’s population size. A healthy TFR oscillates at 2.1 children per woman. Changes in the TFR will affect the demographic distribution of the population. For instance, countries with a TFR below 2.1 may experience an aging population and a reduction in population size.

A population with higher levels of fertility will have lower values in *C(t)*, and *A(t)*, but higher values in *L(t)*. A population with a higher TFR will have a substantial proportion of younger adults in comparison to the older ones, causing a bias in the distribution of age toward the younger ones. Meanwhile, the mortality parameter has a distinct effect on the other parameters. For example, a population with a higher mortality that has an advancement in survival conditions will favor infants and younger children. In that sense, it will promote lower *C(t)* and *A(t)* values. In these communities, decreasing mortality has a similar effect to increasing the fertility rate, and it will promote an increase in the relative size of children that are younger than 5 years (Palloni et al., 2002).

Therefore, population aging is the result of a demographic transition from high to low levels of fertility and mortality. In Latin America and the Caribbean, the shift began in the mid-1960s and continued increasing, leading to rapid population aging. This rapid population aging is also aggravated by a decline in the fertility rate and by an increase in life expectancy. The total fertility rate (TFR) in the Caribbean region went from 2.5 to 2.2 from 2005 to 2020, and by 2050, it is estimated to reach 1.9. However, a different trend is expected for life expectancy. For example, the estimated life expectancy for 2050 in Puerto Rico and Cuba is 84.9 years. Meanwhile, in the Dominican Republic, the estimated life expectancy will be 79.3 years, for the same period. Consequently, adults aged 60 years and older are projected to represent approximately (25%) of the total population in the Spanish-speaking Caribbean, including Puerto Rico, the Dominican Republic, and Cuba (Quashie et al.).

Additionally, outmigration has played a significant role in the aging process in the Caribbean region, especially in Cuba, the Dominican Republic, and Puerto Rico. For example, these three islands have experienced substantial diasporas, mostly to the United States (US). Recently, migration from Puerto Rico to the US increased after hurricanes Irma and Maria, affecting the population’s structure of the Island, as most of the migrants are young people.

Unlike Cuba and the Dominican Republic, Puerto Rico faces a distinct demographic challenge, as its population is aging at a faster rate than almost any other country worldwide, particularly within the Caribbean region (Matos-Moreno et al., 2022a). The age structure of Puerto Rico is evolving due to below-replacement fertility rates, lower mortality rates, and high outmigration among the working-age population (Matos-Moreno et al., 2022a). The outmigration of the working-age adults has placed Puerto Rico in a difficult demographic situation, as it is causing a deficit in the population between 15–54 years, resulting in a phenomenon not observed in other countries, called “aging through compression” (Matos-Moreno et al., 2022a).

According to a study performed by Matos Moreno et al. (2022b), migration is the primary driver of aging in Puerto Rico. Puerto Rico had its first decrease in population, as suggested by recorded history, in 2010. This population decline was caused by decreased birth rates to below replacement levels, and the outmigration of ∼390,507 people between 2000 and 2010; the population went from 3,808,610 to 3,725,789. Consistently, a second migration was reported in the decennial census of 2020, where 439,915 people migrated from the Commonwealth of Puerto Rico, representing an 11.8% reduction in the population. It is estimated that by 2020, Puerto Rico lost thirty-one persons per 1,000 residents, representing a Net Migration Rate (NMR) of −31. The higher negative NMR in Puerto Rico is largely attributable to substantial outmigration from the island.

Furthermore, a report from 2018 by the Puerto Rico Statistics Institute found that 93% of all out migrants in the previous 11 years were below the age of 65, with a median age of 30 years old (Matos-Moreno et al., 2022b). Interestingly, before 2010, the growth of Puerto Rico’s elderly population was similar to that of Cuba and Spain. However, from 2010 to 2020, Puerto Rico set a different pace, and its 65-year-old and older population went from 13.1% to 20.8%, and that change did not occur in Cuba.

According to data from the National Office of Statistics and Information, Cuba in 2024 had a population of 9,901,988 inhabitants distributed across 19 provinces, and a slightly similar panorama to the one in Puerto Rico; it is aging and has a lower total fertility rate (TFR). As stated by the Statistical Health Yearbook of the Ministry of Public Health of Cuba, 25.7% of the population was 60 years and older. Lastly, the birth rate in Cuba, for every 1,000 inhabitants, is 7.2%, and the TFR is 1.29%, and the mortality rate for 2024 by every 1,000 inhabitants was 6.7 for men and 4.3 for women (Ministerio De Salud Pública, 2025).

The Caribbean region has undergone a profound fertility transition since the 1960s, with most countries moving from replacement-level fertility to sustained rates below population replacement over subsequent decades (World Population Review, 2025; Becca et al., 2025). In Puerto Rico, the total fertility rate (TFR) declined from approximately 2.1 children per woman in 2000 to as low as 0.9 in the early 2020s (Becca et al., 2025), with recent estimates indicating a modest increase to 1.18–1.21 births per woman in 2024 (World Population Review, 2025). Similarly, Cuba continues to exhibit one of the lowest fertility rates in the region, with a TFR of approximately 1.3 births per woman, well below replacement level (World Population Review, 2025; Becca et al., 2025). In contrast, the Dominican Republic maintains a comparatively higher fertility rate, exceeding 2.3 children per woman (Becca et al., 2025).

Persistently low fertility, combined with increasing life expectancy, is accelerating population aging across the Caribbean. As younger cohorts continue to shrink and the proportion of older adults expands, the region is experiencing a substantial demographic transformation. In Puerto Rico, projections suggest that the population could decline dramatically by 2050 if fertility remains at current ultra-low levels. More broadly, demographic analyses indicate that adults aged 60 years and older are expected to outnumber individuals younger than 15 years in the coming decades, highlighting the magnitude of the ongoing aging transition (Economic Commission for Latin America and the Caribbean, 2025). Collectively, these trends are reshaping the demographic structure of the Caribbean and creating significant social, economic, and healthcare challenges for the region.

As this Caribbean Hispanic region undergoes rapid population aging, it faces not only significant pressures on its already fragile economies but also a range of health challenges inherently associated with biological aging. Aging is well documented to be linked to a higher prevalence of non-communicable diseases (Palloni et al., 2002). These non-communicable diseases—also referred to as chronic diseases—include cardiovascular disease, hypertension, diabetes, cancer, and chronic respiratory conditions. In the next section, we examine the broader health implications of biological aging among Caribbean Hispanics.

### Biological aging and its implications for chronic diseases in Caribbean Hispanics

Aging is commonly defined as a progressive, generalized impairment of function that results in augmented vulnerability to environmental challenges and a decline in fertility (Kirkwood, 2005). In general, aging is a physiological response that affects most living organisms and is characterized by alterations within different molecular pathways. Aging is also a major risk factor for several non-communicable diseases (Xia et al., 2017). Aging *per se* is a major risk factor for immunosenescence, cardiovascular metabolic disorders, osteoporosis, sarcopenia, arthritis, cataracts, neurodegenerative diseases, and most cancers (Franceschi et al., 2018). The primary cause of many chronic diseases later in life is aging. Based on prior reports, the incidence of age-associated diseases—including Alzheimer’s disease, cardiovascular diseases, and cancer—has been shown to increase markedly with advancing age, doubling every 5 years after the age of 60 (Melzer et al., 2020; Chakravarti et al., 2021).

As the population ages, we anticipate an increased burden of chronic diseases. The Caribbean Hispanic region, with a population that represents 57.6% of the population of the Caribbean, is not excluded from this burden. According to Acosta et al., demographic data from 2021, Cuba, with a population of 11,326,616, Dominican Republic, with 10,847,910, and Puerto Rico, with 3,193,694, have more than 365,000 people living with dementia (Acosta et al., 2021). In Puerto Rico, dementia is the fourth leading cause of death, and eighth in the Dominican Republic. According to the Department of Health from Puerto Rico, Alzheimer’s disease has been the third leading cause of death in women over 60 years since 2018 (Acosta et al., 2021). Similarly, in Cuba, Alzheimer’s disease ranks as the fourth leading cause of death among women and the ninth among men, based on 2021 data from the World Health Organization. In fact, according to the demographic yearbook (Ministerio De Salud Pública, 2025), dementia and Alzheimer’s disease were the sixth cause of mortality for women and men, accounting for 20% of all deaths. Therefore, to address this health issue, the Cuban Government and the Alzheimer’s Research Center announced the National Plan for dementia in 2015. This plan was adopted in 2016 by the Cuban Ministry of Health to increase awareness about the disease, propose strategies to minimize risks, and make efforts to improve diagnosis and access to treatment, among others (Acosta et al., 2021). Likewise, in September 2025, the Department of Health of Puerto Rico developed a Strategic Plan that will be effective from 2026–2030, focusing on addressing health issues associated with Alzheimer’s disease and other dementias. The plan will have different components, with provisions to reduce the risk of developing the disease, improve identification and diagnosis, prevent avoidable hospitalizations, and lastly, provide caregiver support (Departamento de Salud, 2025).

Puerto Rico also has a higher rate of cardiovascular diseases, cancer, and diabetes. In fact, in comparison to Latinos living in the US mainland, Puerto Ricans are more likely to have hypertension, diabetes, and lung disease (Colón-López and García, 2023). Similarly, in the Dominican Republic, chronic diseases had taken a significant toll by 2019, accounting for 72% of all deaths in the Island (Wallace et al., 2023). Lastly, cardiovascular diseases are taking a toll in Cuba, as they are the principal cause of mortality for every 100,000 inhabitants, followed by malignant tumors and cerebrovascular diseases.

Biological aging is characterized by a progressive decline in physiological function that increases vulnerability to disease, environmental stressors, and metabolic dysregulation. These age-related changes arise from alterations in key molecular and cellular pathways, which increase susceptibility to chronic conditions. For instance, the inflammation pathway is one of the most disrupted mechanisms in the aging process, driven by immune system dysregulation, cellular damage, and mitochondrial dysfunction.

Although substantial advances have been made in elucidating the biological mechanisms linking aging, inflammation, and chronic diseases, Caribbean Hispanics remain largely underrepresented in this body of research. Therefore, addressing the existing gaps in knowledge regarding inflammation, biomarkers, therapeutic interventions, and disease mechanisms will be essential to improving prevention, diagnosis, and treatment in this population.

### Research gaps among Caribbean Hispanics

Despite research involving Caribbean Hispanics having expanded in recent years, the population remains markedly underrepresented in studies evaluating biomarkers, anti-aging therapeutic interventions, and precision medicine approaches. Consequently, several critical research priorities remain unresolved. Four major gaps emerge from the current literature review. First, there is a lack of large-scale clinical trials evaluating anti-inflammatory, disease-modifying, or microbiome-based therapies specifically in Caribbean Hispanics. Second, although inflammatory pathways are increasingly recognized as key contributors to AD pathogenesis, more studies are needed to evaluate inflammatory biomarkers to predict treatment response or guide precision medicine approaches in this population. Third, the role of non-coding RNAs (ncRNAs), including microRNAs, as therapeutic targets or biomarkers remains largely unexplored among Caribbean Hispanics despite their known involvement in neuroinflammatory and neurodegenerative processes. Finally, limited longitudinal studies have examined how genetic susceptibility factors, inflammatory burden, gut microbiome composition, and social determinants of health interact to influence disease progression and therapeutic outcomes.

Addressing these gaps will be essential for advancing precision medicine initiatives in Caribbean Hispanics. Future studies should prioritize the inclusion of diverse Hispanic populations in clinical trials, the validation of population-specific biomarkers, and the development of targeted interventions that address the unique genetic, biological, and environmental factors influencing AD risk and progression in the Caribbean region.

Lastly, we want to emphasize this population not only because there is a research gap, but there is a heightened vulnerability of Caribbean Hispanics due to accelerated demographic aging, socioeconomic inequities, and accelerated biological aging through weathering processes, which collectively may increase the burden in this group to age-related diseases. Therefore, this population could potentially be an ideal population to benefit from novel anti-aging strategies or interventions.

### Weathering hypothesis

The weathering hypothesis was first proposed by Arline T. Geronimus to explain racial disparities in fertility patterns and birth outcomes in the United States (Geronimus, 1992). Geronimus observed that, unlike non-Hispanic White infants, African American infants exhibited higher survival rates when born with teen mothers. In contrast, birth outcomes for African American women declined more steeply with advancing maternal age, resulting in a higher mortality rate at older maternal ages than at younger ages. This pattern diverged from the well-documented curvilinear association between maternal age and birth outcomes typically seen in the general population, where teenage mothers and women over age 30 are at highest risk, and outcomes are most favorable for mothers in their mid-20s (Forde et al., 2019).

The weathering hypothesis posits that these differences reflect the cumulative impact of chronic exposure to social, economic, and environmental adversity, including racial discrimination, structural inequities, daily slights, and threats to identity (Simons et al., 2021; Geronimus et al., 2016). Such exposures accelerate biological aging processes, resulting in an earlier onset of health deterioration among disadvantaged groups relative to more advantaged populations of the same chronological age (Forde et al., 2019; Simons et al., 2021). In this framework, “weathering” describes the physiological wear and tear produced by persistent stressors across the life course, which manifest in elevated rates of illness, disability, and adverse reproductive outcomes.

To empirically evaluate this concept, Geronimus and colleagues expanded on allostatic load—a composite measure of cumulative physiological dysregulation across multiple biological systems—as an indicator of weathering, building on McEwen and Stellar’s 1993 concept describing the physiological burden of chronic stress and life events (Melzer et al., 2020; Chakravarti et al., 2021). Higher allostatic load scores among African Americans at younger ages, compared with their White counterparts, provide evidence that the burden of structural racism and socioeconomic disadvantage contributes to premature health decline (Forde et al., 2019; Simons et al., 2021; Geronimus et al., 2006). More recent studies extend the weathering framework beyond birth outcomes to broader health domains, linking it to chronic diseases, inflammation, and mortality disparities observed in marginalized populations (Simons et al., 2021).

Although originally formulated to explain disparities among African Americans, the weathering framework has increasing relevance for Caribbean Hispanics, including Puerto Ricans, Dominicans, and Cubans, both in the Caribbean and in diaspora communities in the United States. These populations often face intersectional disadvantages, such as socioeconomic marginalization, structural racism, migration-related stress, and limited access to healthcare. For example, Puerto Rican women living in the mainland U.S. have been shown to experience elevated rates of adverse birth outcomes compared to non-Hispanic White women, patterns that echo weathering-related dynamics. Similarly, Caribbean Hispanics often display higher prevalence of chronic conditions such as hypertension, diabetes, and obesity at earlier ages compared with more advantaged groups, suggesting that the cumulative stress of disadvantage may produce a weathering effect in these populations as well. Expanding the weathering hypothesis to Caribbean contexts underscores the importance of examining how structural inequities, colonial histories, and ongoing migration patterns accelerate aging processes and health disparities across racialized and ethnic minority groups in the Americas.

While the weathering hypothesis has been highly influential in explaining how chronic exposure to structural disadvantage becomes biologically embodied over the course of life, it is important to acknowledge its limitations. Some scholars have cautioned against interpretations that could inadvertently imply biological determinism by overemphasizing physiological deterioration without sufficient attention to the dynamic social processes that shape vulnerability and adaptation. Rather than suggesting an inevitable trajectory of decline, contemporary applications of the weathering framework emphasize that biological responses to adversity are socially patterned and potentially modifiable through structural interventions and psychosocial resources. Emerging evidence highlights the role of resilience factors—including social support, community cohesion, cultural identity, religious engagement, and adaptive coping strategies—in buffering the health consequences of chronic stress and discrimination. These protective influences may mitigate allostatic burden and help explain heterogeneity in health outcomes among individuals and communities exposed to similar stressors. Thus, weathering should be understood not as a deterministic model of biological inevitability, but as a life-course framework that situates biological processes within broader social contexts, recognizing both the cumulative effects of structural inequities and the capacity for resilience in the face of adversity (Geronimus, 1992; Forde et al., 2019; Geronimus, 2023; McDonough et al., 1982).

### The allostatic load as a marker of weathering

The allostatic load results from the cumulative effect of experiences through the lifespan, including subtle and long-standing situations and major life events (Gu et al., 2021). It also includes the physiological ramifications of unhealthy habits such as poor sleep and circadian disruption, lack of exercise, smoking, alcohol consumption, and an unhealthy diet (Chakravarti et al., 2021).

The term allostatic derives from the definition of allostasis, which refers to the capability of an organism to achieve stability through change. When its ability to achieve stability is exceeded, allostatic overload is produced. During allostatic overload, the stress response system is repeatedly activated, and the buffering factors are not properly functioning.

The allostatic load or allostatic overload could be triggered by different scenarios: (a) exposure to persistent stressors, (b) lack of adaptation to repeated stressors, (c) inability to shut off the stress response after the stressor is not present, and lastly (d) the allostatic response is not enough to deal with the stressor. A physiological response to an allostatic load involves the integration of different physiological systems at various levels. For example, the neuroendocrine and immune systems are mobilized in response to internal and external challenges, facilitating adaptation to stressors and environmental threats. The brain and its neurochemical orchestra are affected by both genomics and nongenomic mechanisms. Lastly, the immune system suffers and undergoes adjustments with immunosuppressive effects overall.

Consistent with the weathering argument, Black Americans showed higher levels of allostatic load than white people, and this finding continues to be significant after adjusting for poverty (Simons et al., 2021). These findings were comparable to other studies performed by McDade and associates, in which inflammation was used as a biomarker (Ferrucci and Fabbri, 2018).

Nowadays, researchers use the Weathering hypothesis as a framework for racial and ethnic health disparities in minority populations, particularly African Americans and Hispanics. Today, this hypothesis is used to elucidate how social status and inequalities might contribute to and produce health disparities later in life (Schmeer and Tarrence, 2018).

### Role of aging in inflammation, cancer, and neurodegeneration

Inflammaging refers to the chronic low-grade inflammatory state that accompanies aging and contributes to the progressive decline of physiological function and increased susceptibility to age-related diseases, including cancer and neurodegenerative disorders (Li et al., 2023; García-Domínguez, 2025; Franceschi et al., 2000). There is a well-established relationship between chronic inflammation and age-associated diseases, such as cancer and neurodegeneration (Baechle et al., 2023). During chronic inflammation, there is an increase in the levels of inflammatory markers such as cytokines, such as IL-6, TNF-α, and IL-1β, as well as the activation of NF-κβ, which is associated with the expression of inflammatory genes. Table 1 summarizes the associations of these cytokines with low-grade chronic inflammation and aging, as well as their connections to diseases such as cancer and neurodegeneration.

**TABLE 1:** Key pro-inflammatory cytokines linking inflammaging, aging, cancer, and neurodegeneration.

Cytokine	Association of inflammation and aging	Role in cancer	Role in neurodegeneration	References
IL-6	Rises progressively from mid-life; increases systemic low-grade inflammation and drives acute-phase protein (CRP) synthesis.	Sustains tumor-cell survival, invasion, and metastasis.	Enhances microglial activation and is linked to faster memory decline and brain atrophy in Alzheimer’s disease models.	Franceschi et al. (2018), Li et al. (2023), Rašková et al. (2022), Lyra et al. (2021)
TNF-α	The principal component of low-grade inflammation signs, chronic elevation, promotes weakness and immunosenescence.	Potentiates angiogenesis, tumor growth, and metastatic spread.	Microglial TNF-α drives dopaminergic neuronal death in Alzheimer’s disease, Parkinson’s disease, and other CNS disorders.	Rodrigues et al. (2021), Gao et al. (2023), Dash et al. (2021)
IL-1β	Age-related NLRP3 inflammasome activation enhances systemic IL-1β production, thereby reinforcing low-grade inflammation.	Promotes tumor angiogenesis and intravasation.	Significant mediator of inflammasome-driven neuroinflammation; excess IL-1β accelerates neuronal injury in Alzheimer’s models.	Li et al. (2023), Shive and Pandiyan (2022), Ikanga et al. (2024), McGroarty et al. (2025)

Recent research has further elucidated the link between inflammation and age-associated diseases, including cancer (Baechle et al., 2023) and neurodegenerative disorders such as Alzheimer’s disease (Gonzales et al., 2022) temporal frontal dementia, and Parkinson’s. Chronic inflammation can create a pro-tumorigenic environment, promote genomic instability, and impair neuronal function, serving as a shared mechanism underlying both cancer and neurodegeneration. Inflammaging plays a significant role in cancer development and progression. Cellular senescence contributes to inflammaging through the accumulation of senescent cells and their senescence-associated secretory phenotype (SASP) factors, which can remodel the tissue microenvironment and promote disease-associated inflammation (Dong et al., 2024). In cancer, low-grade chronic inflammation and senescence-associated signaling can create a pro-tumorigenic microenvironment by supporting genomic instability, angiogenesis, immune modulation, and tissue remodeling. Clinical observations, such as the increased risk of colorectal, prostate and lung cancer in elderly Caribbean populations, where older adults account for over half of all new cancer cases, highlight the role of chronic inflammation as a fertile ground for tumorigenesis (Pilleron et al., 2019).

Similarly, inflammaging is a critical contributor to neurodegenerative diseases. In neurodegenerative diseases, inflammaging may exacerbate pathological processes by sustaining neuroinflammatory signaling, oxidative stress, mitochondrial dysfunction, and impaired autophagy. This persistent inflammatory response exacerbates hallmark pathologies of neurodegeneration, including amyloid-β accumulation in Alzheimer’s disease and dopaminergic neuronal loss in Parkinson’s disease. Furthermore, mitochondrial dysfunction and impaired autophagy in aged neurons further promote the inflammatory cascade, linking cellular senescence to progressive neuronal decline. These findings support the concept that inflammaging serves as a molecular bridge between aging, cancer, and neurodegeneration. Because cellular senescence is a major contributor to inflammaging, the next section focuses on how senescent cells and SASP factors sustain age-related inflammation. Therefore, understanding the interplay between cellular senescence and inflammaging is crucial for identifying therapeutic strategies to disrupt the vicious cycle that accelerates age-related pathologies.

### Hallmarks of aging

Human biomarkers such as blood biomarkers (e.g., glucose, LDL-cholesterol, HDL-cholesterol, albumin) and anthropomorphic measures are of major importance for medicine and biomedical research (Le Goallec and Patel, 2019). Biomarkers are useful for diagnosing diseases, evaluating treatments, predicting clinical outcomes, and serving as surrogates for clinical endpoints. Biomarkers can also be used to identify a specific patient population, and to enrich a patient population that is expected to be more sensitive to a drug (Wickström and Moseley, 2017).

According to the United Nations projections, the number of people aged 60 years and over is growing; estimates suggest that this population was 1.4 billion in 2017 and is expected to increase to 2.1 billion by 2050 (Rodrigues et al., 2021). As the population worldwide is getting older, scientists are promoting efforts to understand aging and its main key drivers.

**FIGURE 1:**
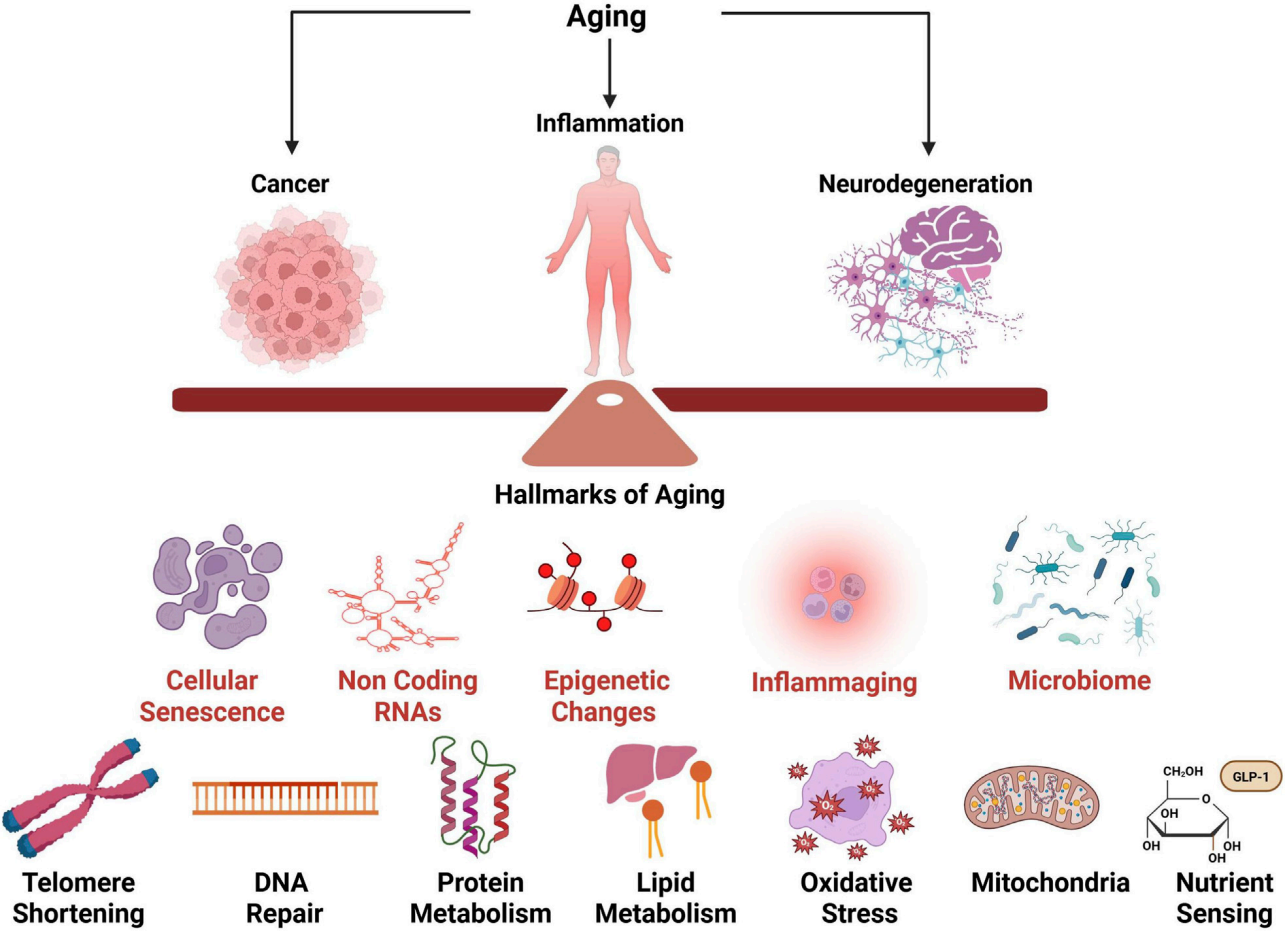
Aging, chronic low-grade inflammation, cancer, neurodegeneration, and the hallmarks of aging. Aging is associated with a progressive increase in chronic low-grade inflammation, known as inflammaging, which contributes to biological dysregulation and acts as a molecular link between age-related diseases such as cancer and neurodegeneration. This figure illustrates how aging and inflammation are interconnected with multiple hallmarks of aging, including cellular senescence, non-coding RNAs, epigenetic changes, microbiome alterations, telomere shortening, impaired DNA repair, altered protein and lipid metabolism, oxidative stress, mitochondrial dysfunction, and dysregulated nutrient sensing. Together, these mechanisms create a biological environment that promotes tissue dysfunction, tumorigenesis, and neuronal damage, highlighting inflammation as a point of intersection between cancer and neurodegenerative diseases. Created in BioRender. Santiago, M. (2025) https://BioRender.com/undefined.

Therefore, the American Federation of Aging Research has defined the criteria for a true biomarker of aging. The following criteria were proposed: 1) can model the rate of aging, 2) represent a core biological process of healthy aging, 3) can be tested in humans without harm, and 4) can be tested in humans and animal models. Several markers have been proposed since 2013; however, not all the biomarkers will fully comply with the previous criteria (Xia et al., 2017). Several studies have identified key hallmarks associated with aging, including telomere shortening, DNA repair mechanisms, epigenetic modifications, transcriptomic profiles, non-coding RNAs, microbiome, nutrient sensing, protein and lipid metabolisms, oxidative stress and mitochondria, cell senescence and chronic inflammation (Figure 1: Aging, Chronic Low-Grade Inflammation, Cancer, Neurodegeneration, and the Hallmarks of Aging) (Xia et al., 2017; Rodrigues et al., 2021; Vaiserman and Krasnienkov, 2021; Pal and Tyler, 2016; la Torre et al., 2023).

The microbiome has emerged as an important modulator of aging and multiple pathologies associated with it. Its contribution to host defense against pathogens, nutrient metabolism, and the generation of bioactive compounds, such as vitamins, amino acid derivatives, bile acids, and fatty acids, is essential for maintaining organismal homeostasis (López-Otín et al., 2023). However, age-related changes can alter both its composition and function, thereby promoting intestinal dysbiosis. This loss of microbiota–host balance has been linked to chronic inflammation and physiological alterations affecting multiple organs and systems, including the central nervous system. The relevance of this phenomenon has driven the development of studies in animal models and human cohorts, which have generated clinical and molecular evidence supporting the role of the microbiome in systemic health.

In this review, we emphasize biological markers with strong potential for the development of interventions targeting aging and inflammation. Particular attention is given to epigenetic changes, cellular senescence, and inflammaging, which have emerged as promising therapeutic targets due to their fundamental roles in age-related physiological decline and disease susceptibility.

### Epigenetic changes

Epigenetic changes in general refer to those changes that are heritable but, unlike mutations, do not affect the sequence of DNA. Although the chromosomes in our genome convey genetic information, the epigenome is responsible for its use and stability, connecting the genotype with the phenotype (Pal and Tyler, 2016). In monozygotic twins, genetic factors account for only about 20%–30% of the differences in lifespan, while the remaining variation is largely attributed to epigenetic drift that occurs throughout life. The epigenetic changes are distinctive mechanisms, irreversible, and include DNA methylation, chromatin remodeling, histone modification, and noncoding RNA regulation (microRNAs) (Gonzales et al., 2022).

DNA methylation is the most studied and best understood of epigenetic changes. It happens at the DNA level, and its key role is to regulate gene expression. Its mechanism of action involves the recruitment of proteins required for gene silencing, or it prevents the interaction between DNA and transcription factors. For example, DNA methylation is important during X chromosome inactivation, genomic imprinting, and repression of retroviral elements (Gonzales et al., 2022).

While DNA methylation is associated with the regulation of transcriptional initiation activity, DNA hypomethylation results in higher transcriptional activity. These patterns of DNA methylation have been suggested to change as a function of age. Therefore, changes in DNA methylation at CPG sites are being used to generate algorithms for comparing the chronological age with the biological age of an individual. Today, different algorithms termed “epigenetic clocks” have been developed for human assessment and also for mouse models (Petkovich et al., 2017; Wang et al., 2017; Kabacik et al., 2022).

Lastly, other epigenetic changes are chromatin remodeling and chromatin heterogeneity; these two also increase with age. Conversely, histone acetylation tends to decrease with age, resulting in a more tightly packaged DNA structure, causing changes in gene expression (Gonzales et al., 2022). For this review, we will not discuss them; however, more literature can be found in the following literature (Gonzales et al., 2022; Pal and Tyler, 2016).

## Non-coding RNAs

Non-coding RNAs (ncRNAs) are a group of RNAs that do not encode proteins but play important roles in regulating protein synthesis in other genes. Among the ncRNAs, siRNA and miRNA are the most extensively explored. Studies have shown that siRNA, miRNA, and other ncRNAs play an important role in epigenetic regulation (Wei et al., 2017). One characteristic they share is that both inhibit gene expression at a post-transcriptional stage, acting on messenger RNA (mRNA) before it is converted into protein. According to the central dogma of molecular biology, gene expression requires that mRNA be first synthesized, then exported from the nucleus, and finally translated into protein by the ribosome. Both siRNA and miRNA act in the cytoplasm after the mRNA exits the nucleus and before it arrives at the ribosome to be translated. This mechanism of action allows inhibition of the message a gene intends to express before it is translated into protein. A detailed discussion of the differences between the mechanisms of action of siRNAs and miRNAs, as well as the factors underlying these distinctions, is beyond the scope of this review. For comprehensive overviews of their respective mechanisms and key differences, readers are referred to the following references (You et al., 2019; Lam et al., 2015).

One of the major differences between siRNA and miRNA is that most miRNAs bind to the 3′UTR of their target mRNAs through partial pairing. This led to more off-target effects, less specificity, making them poor candidates for treatment development. Conversely, siRNAs act through a very similar mechanism; however, a key distinction is that they pair with perfect complementarity to a single mRNA target, enabling highly specific gene silencing (Lam et al., 2015). This provides the opportunity to specifically inhibit a gene of interest by targeting its mRNA. For this reason, siRNA is more extensively studied than miRNA, as siRNA provides higher specificity as a therapeutic strategy for single-gene targeting (Leng et al., 2020). Consequently, FDA approved therapies are based on siRNA and not miRNA. Recently, in March 2025, the FDA approved a novel siRNA therapy, adding to the only six siRNA drugs ever approved (each within the past decade) and underscoring the expanding potential of this technology across new therapeutic areas. These FDA-approved siRNA therapies now include Patisiran, Givosiran, Lumasiran, Inclisiran, Vutrisiran, Nedosiran, and the most recent addition, Fitusiran (Supplementary Table S1) (U.S. Food and Drug Administration, 2025a; U.S. Food and Drug Administration, 2025b; U.S. Food and Drug Administration, 2025c; U.S. Food and Drug Administration, 2025d; U.S. Food and Drug Administration, 2025e; U.S. Food and Drug Administration, 2025f; U.S. Food and Drug Administration, 2025g).

### The role of siRNA and miRNA in aging

We first searched for preclinical studies demonstrating a relationship between siRNA, miRNA, and aging, and found evidence that siRNA can influence aging-related pathways in preclinical models. In one study, researchers used siRNA to knock down Silent Information Regulator 6 (SIRT6), a longevity-associated factor with protective roles against aging-related cellular dysfunction. Silencing Sirt6 increased cellular senescence, as shown by elevated SA-β-gal positivity, and upregulated plasminogen activator inhibitor-1 (PAI-1), a well-known marker and mediator of senescence (Aobulikasimu et al., 2023). SA-β-gal and PAI-1 are both potent senescence markers. In addition to these findings, pre-clinical evidence suggests that miRNA also plays a key role in the gene Silent information regulator 1 (*SIRT1*). This gene has been shown to play an essential role in regulating cell cycle, senescence, and metabolism. The authors identified *SIRT1* as a direct target of the miRNA miR-34a. MiR-34a has been shown to regulate apoptosis and contribute to aging-related processes. To confirm this interaction, they inhibited *SIRT1* both by overexpressing miR-34a and by using a siRNA specific for *SIRT1*. In both approaches, the results were consistent; *SIRT1* suppression led to increased apoptosis and enhanced p53 acetylation, highlighting that loss of *SIRT1* activity promotes senescence-like changes. This demonstrates that miR-34a exerts its pro-aging effects largely by targeting *SIRT1*, and siRNA knockdown validates this pathway (Zhang et al., 2015).

Taken together, these findings illustrate preclinical studies on how both siRNA and miRNA can modulate key regulators of cellular aging, either by directly silencing protective genes such as *SIRT6* and *SIRT1*, or by indirectly exerting effects through miRNA–gene interactions. To provide a clearer overview, we compiled tables summarizing aging-related genes experimentally targeted by siRNA (Table 2) and miRNA (Table 3), as well as gene interactions implicated in aging. However, these findings should be interpreted as mechanistic and hypothesis-generating rather than clinically established evidence. Most of the available studies were conducted in cell culture systems or animal models, and their applicability to human aging, stress-related health disparities, and Caribbean Hispanic populations remains uncertain. For simplicity, studies using shRNA are included together with siRNA, as both act through the same RNA interference pathway (Alshaer et al., 2021). For simplicity, studies using shRNA are included together with siRNA, as both act through the same RNA interference pathway (Alshaer et al., 2021).

**TABLE 2:** Aging-related genes inhibited with siRNA/shRNA.

Gene	Gene/Pathway context reported in the study	Effect of siRNA inhibition	References
Sirtuin 6 (*SIRT6*)	SIRT6 regulates osteocyte senescence via the PAI-1 pathway; its deficiency accelerates bone metabolic disruption associated with aging.	siRNA knockdown of *SIRT6* using the sequence 5′-UUAU UGCAU UGGG AGCUU CUG-3′ increased *SOST*, *FGF23*, *PAI-1*, *p16*, and *IL-6* expression in osteocyte-like cells, promoting cellular senescence.	Aobulikasimu et al. (2023)
Programmed cell death protein 4 (*PDCD4*)	PDCD4 acts as a tumor suppressor in hepatoma cells; its loss induces cellular senescence through upregulation of p21.	siRNA knockdown of *PDCD4* using effective sequences (5′-GCA​CAA​CUG​AUG​UGG​AAA​A-3′ and 5′-GUG​UUG​GCA​GUA​UCC​UUA​G-3′) induced senescence-like growth arrest through RB/CDK pathways by up-regulating *p21* expression in human hepatoma cells.	Guo et al. (2019)
Splicing factor 3B subunit 4 (*SF3B4*)	SF3B4 regulates cellular senescence through RNA splicing; its depletion promotes senescence and suppresses therapy-induced senescence in cancer cells.	siRNA knockdown of *SF3B4* using the sequence (5′-GCA​GUA​CCU​CUG​UAA​CCG​U-3′) induced senescence markers in human fibroblasts (SA-β-gal, p21, SASP genes).	Yang et al. (2024)
Secreted frizzled-related protein 4 (*SFRP4*)	SFRP4 antagonizes Wnt signaling in dermal fibroblasts; its upregulation during aging promotes SASP expression, reduces collagen III synthesis, and contributes to skin aging.	siRNA knockdown of *SFRP4* using commercial Silencer™ pre-designed siRNAs (#121422–121424, Thermo Fisher Scientific) reduced SASP factors (IL-1α, IL-6, IL-8, MMP3, TNF-α) and increased collagen III expression in aged fibroblasts.	Takaya et al. (2022)

**TABLE 3:** MiRNA–gene interactions implicated in aging.

miRNA and Target(s)	Role of the target in aging	Functional effect of miRNA	References
miR-29a - Serpin family H member 1 (SERPINH1)	miR-29a was upregulated in aged mouse hearts; SERPINH1 was identified as its direct target involved in collagen processing and ECM remodeling.	Age-related increase in miR-29a suppresses SERPINH1, reducing collagen, cell migration, and proliferation suggesting anti-fibrotic adaptation.	Rusu-Nastase et al. (2022)
miR-302b – Cyclin-dependent kinase inhibitor 1A (CDKN1A)	CDKN1A (p21) was identified as a direct target of miR-302b in senescent cells; its accumulation was associated with proliferative arrest in aged mice.	Exosomal miR-302b suppresses Cdkn1a and Ccng2, reversing proliferative arrest of senescent cells and inducing rejuvenation in aged mice.	Bi et al. (2025)
miR-21-5p – Programmed cell death 4 (PDCD4)	PDCD4 was identified as a validated target of miR-21-5p; its downregulation was associated with increased IL-6 and TNF-α production in the context of inflammaging.	Upregulation of miR-21-5p suppresses PDCD4 promoting the expression of the two major proinflammatory cytokines that promote cellular senescence (IL-6, TNF-α).	Olivieri et al. (2021)
miR-146a – Interleukin-1 receptor-associated kinase 1 (IRAK1)	IRAK1 was identified as a validated target of miR-146a within the NF-κB signaling pathway; its overactivation was linked to chronic low-grade inflammation in aging.	miR-146a reduces the expression of IRAK1 a key activator of the NF-κB pathway. By doing this, it helps control excessive inflammation that contributes to inflammaging.	Olivieri et al. (2021)
miR-124 – Ryanodine receptor 3 (RyR3)	RyR3 was identified as a direct target of miR-124 in hippocampal neurons; its derepression in aged mice was associated with disrupted intracellular calcium signaling.	Decreased miR-124 expression with age leads to higher RyR3 levels, causing abnormal calcium signaling, synaptic dysfunction, and memory impairment.	Liu et al. (2022)

The findings described above highlight that ncRNAs, particularly siRNAs and miRNAs, are key modulators of molecular pathways associated with aging. These ncRNAs undergo changes in expression and epigenetic regulation during aging (Zhang et al., 2015). Notably, whether these RNA-mediated mechanisms causally contribute to aging in humans or instead reflect downstream consequences of senescence and chronic inflammation remains an open question. Although their roles in aging are increasingly supported by preclinical evidence, with emerging but still limited support from human observational and clinical studies, it is important to place these findings within the broader context of inflammaging and the biological processes underlying age-related decline. In line with this, emerging evidence indicates that specific senescence-associated miRNAs, including members of the miR-17 family, may regulate key pathways involved in cell-cycle arrest and cellular aging. For example, miR-93 and miR-20a are members of the miR-17 family. Both miRNAs are reduced in senescent mesenchymal stem cells (MSCs), leading to upregulation of p21, a key mediator of cell-cycle arrest (Alshaer et al., 2021). Likewise, miR-195 has been found to promote senescence by suppressing both SIRT1 and TERT, two genes essential for DNA repair, telomere integrity, and cellular longevity, whereas miR-486-5p induces a premature senescence phenotype by inhibiting SIRT1 expression (Alshaer et al., 2021). Collectively, these preclinical findings suggest that ncRNAs play a significant role in the regulation of senescence, reinforcing their potential applications in aging research and their importance in understanding the molecular mechanisms driving age-related decline (Sherazi et al., 2023).

Beyond their direct involvement in cellular senescence, ncRNAs may also contribute to aging through their interaction with chronic inflammatory and epigenetic networks. This is particularly relevant because inflammaging is not driven by a single molecular pathway, but rather by the convergence of cellular senescence, immune dysregulation, epigenetic remodeling, and environmental factors such as the gut microbiota. Consistent with this view, recent studies conducted in 2025 have shown a bidirectional relationship between gut microbiota and epigenetic regulation, including ncRNA-related mechanisms, in intestinal diseases such as inflammatory bowel disease and colorectal cancer (Lin et al., 2025).

These findings highlight the importance of microbiota-driven epigenetic dysregulation and its contribution to chronic inflammatory states. Along the same line, similar ncRNA alterations may also be relevant in Alzheimer’s disease, where neuroinflammation is a major pathogenic feature. Although these mechanisms have been more extensively investigated in intestinal diseases, they may represent promising avenues for future research in AD. However, direct evidence linking microbiota-driven ncRNA dysregulation to AD pathogenesis remains limited. Supporting this hypothesis, recent evidence in Puerto Rican aging Hispanics with Alzheimer’s disease has shown gut microbiota dysbiosis associated with cognitive impairment severity, suggesting that microbiome alterations may contribute to disease progression (Verdoorn et al., 2021) This observation can be linked to the growing literature on ncRNAs, particularly miRNAs, which are increasingly recognized as mediators of host–microbiota communication within the microbiota–gut–brain axis (Potter et al., 2021). In this context, miRNAs are of special interest not only because of their regulatory role in inflammatory and neurodegenerative pathways, but also because of their potential as biomarkers of disease status, progression, and severity (Potter et al., 2021). These findings are significant for Hispanic populations, specifically Puerto Ricans with Alzheimer’s disease, because ncRNA-based approaches may help refine biomarker discovery and inform future therapeutic strategies in this understudied population.

Taken together, these findings suggest that ncRNAs are closely connected to the molecular networks that regulate inflammation, senescence, and age-associated decline. The link between ncRNA regulation and inflammaging is particularly significant, since miRNAs and siRNAs can directly modulate pro-inflammatory mediators such as cytokines and inflammasome components in preclinical studies (Zhang et al., 2015). By influencing these pathways, ncRNAs may act not only as biomarkers but also as conceptually promising therapeutic targets in age-associated inflammation (Zhang et al., 2015). However, these findings remain largely preclinical and should be interpreted as hypothesis-generating rather than clinically established evidence. Further studies in human cohorts, particularly in Caribbean Hispanic populations, are needed to determine whether ncRNA-related mechanisms contribute to aging, inflammaging, and stress-related health disparities in a population-specific context. Therefore, understanding how ncRNAs shape inflammaging and senescence provides an important foundation for discussing the cellular processes that drive aging, particularly cellular senescence and its associated inflammatory phenotype. Taken together, these findings suggest that ncRNAs are closely connected to the molecular networks that regulate inflammation, senescence, and age-associated decline. The link between ncRNA regulation and inflammaging is particularly significant, since miRNAs and siRNAs can directly modulate pro-inflammatory mediators such as cytokines and inflammasome components in preclinical studies (Olivieri et al., 2021). By influencing these pathways, ncRNAs may act not only as biomarkers but also as conceptually promising therapeutic targets in age-associated inflammation (Olivieri et al., 2021). Therefore, understanding how ncRNAs shape inflammaging and senescence provides an important foundation for discussing the cellular processes that drive aging, particularly cellular senescence and its associated inflammatory phenotype.

### Cellular senescence as a driver of inflammaging and age-related disease

Cellular senescence is a state of stable cell cycle arrest induced by various stressors, including telomere shortening, oxidative stress, DNA damage, and oncogene activation (Hernandez-Segura et al., 2018). Although senescent cells no longer proliferate, they remain metabolically active and undergo profound changes in gene expression, morphology, and secretory function (Gorgoulis et al., 2019). These cells are characterized by the secretion of a hallmark collection of proinflammatory cytokines, chemokines, growth factors, and proteases, collectively referred to as the senescence-associated secretory phenotype (SASP) (Birch and Gil, 2020). The release of cytoplasmic chromatin fragments and mitochondrial DNA, together with NF-κB signaling and the cofactor C/EBPβ, are important factors in SASP activation (Zhang et al., 2022). Through the persistent secretion of SASP factors, senescent cells contributea chronic pro-inflammatory environment that not only contributes to aging but also serves as a key driver of the onset and progression of age-related diseases such as cancer and neurodegeneration.

In cancer, SASP factors such as IL-6 and TNF-α activate the NF-κB and STAT3 pathways, promoting tumor progression (Baechle et al., 2023; Birch and Gil, 2020). In neurodegeneration, senescent glial and neuronal cells amplify neuroinflammation, contributing to synaptic dysfunction and neuronal loss (Zhang et al., 2022). There are both molecular and phenotypic biomarkers associated with aging and the accumulation of senescent cells. Among the molecular biomarkers, senescence-associated β -galactosidase (SAβ-gal) is particularly prominent, as its activity increases with aging (Yasuda et al.). This activity reflects enhanced lysosomal function, which may contribute to cellular deterioration and damage associated with cancer and neurodegeneration (Sikora et al., 2021). Additional molecular markers include the proteins p16^INK4a^, p53, and p21^CIP1^, which participate in the induction of cell cycle arrest (de Mera-Rodríguez et al., 2021). According to Xia et al. (2017), phenotypic biomarkers include the activation and persistence of the DNA damage response, telomere shortening, and circulating levels of pro-inflammatory cytokines such as IL-6, all of which have been proposed as indicators of biological aging and its pathological consequences, closely linked to inflammatory processes and altered intercellular communication. Cellular senescence and low-grade chronic inflammation are increasingly recognized as contributors to cancer and neurodegeneration, although their effects are highly context dependent. The presence of these biomarkers and inflammatory mechanisms highlights cellular senescence as an important contributor to inflammaging and age-related disease. These observations support therapeutic approaches aimed at targeting senescent cells or modulating SASP activity. Based on this rationale, the following section discusses senolytic and senomorphic strategies as emerging approaches to target cellular senescence and SASP-mediated inflammation.

### Senolytics and senomorphic as a therapeutic strategy

Senolytics are an emerging class of natural and synthetic compounds designed to selectively eliminate senescent cells, thereby reducing senescence-associated secretory phenotype (SASP) mediated inflammation and potentially modifying the progression of certain age-related diseases (Kirkland and Tchkonia, 2020; Saliev and Singh, 2025; Luís et al., 2022). However, most of the supporting evidence for senolytic interventions in the context of stress-related or social adversity–associated aging is preclinical. Human trials remain limited, and none have specifically evaluated effects in Caribbean Hispanic populations.

Although senolytic therapies have demonstrated encouraging effects in preclinical models of aging and age-related diseases, most of the available evidence comes from animal studies, limiting direct clinical translation. Consequently, current evidence should be interpreted cautiously, and senolytics cannot yet be considered a validated therapeutic intervention for human aging as their clinical efficacy and long-term safety remain uncertain.

Senolytic strategies are designed to selectively eliminate senescent cells, which undergo stable cell-cycle arrest but persist in tissues by activating senescent cell anti-apoptotic pathways (SCAP) (Kirkland and Tchkonia, 2020; Kirkland and Tchkonia, 2017). By inhibiting SCAP, senolytics preferentially induce apoptosis in senescent cells while sparing normal, proliferating, or quiescent cells. This targeted clearance not only alleviates tissue dysfunction but also reduces the secretion of SASP, a complex mixture of pro-inflammatory cytokines, chemokines, proteases, and growth factors that drive chronic inflammation and contribute to cancer, neurodegeneration, and other age-related pathologies. Research has identified several categories of senolytic agents, including natural compounds, synthetic drugs, and pharmacological complexes. These agents act through multiple anti-apoptotic resistance pathways, including inhibition of the BCL-2 family, the PI3K/AKT pathway, NF-κB signaling, and other SCAP related mechanisms, promoting the selective elimination of senescent cells while preserving normal tissue function (Aobulikasimu et al., 2023).

**FIGURE 2:**
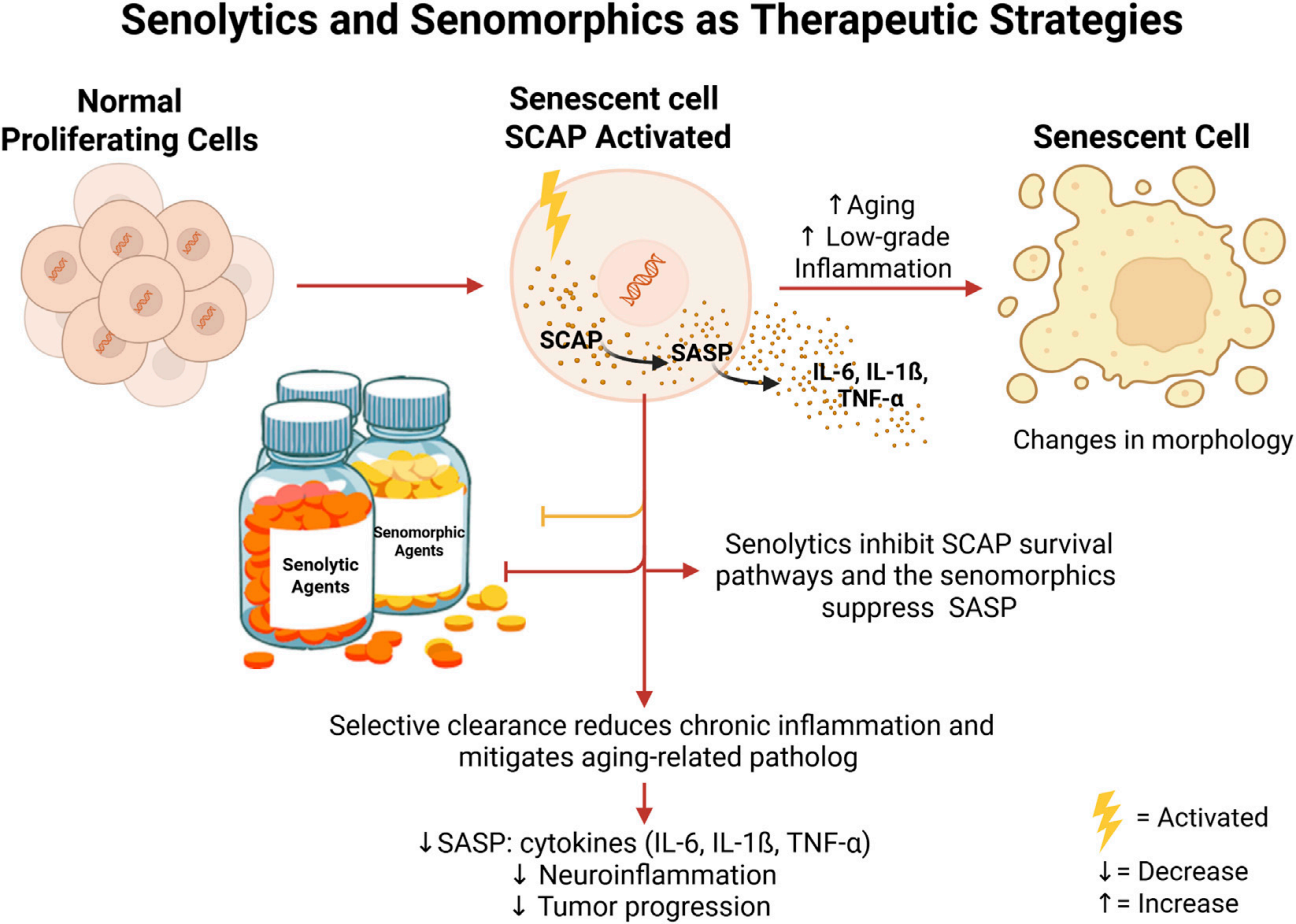
Overview of senolytic and senomorphic therapeutic strategies targeting cellular senescence. Normal cells proliferate in a characteristic manner; however, under certain stimuli, cells may activate senescent cell anti-apoptotic pathways, known as SCAPs, which promote survival despite cellular damage or stress. This activation contributes to the secretion of the senescence-associated secretory phenotype (SASP) and morphological changes that characterize senescent cells. This figure demonstrates how aging and chronic low-grade inflammation promote the accumulation of senescent cells, leading to the release of pro-inflammatory cytokines such as IL-6, IL-1β, and TNF-α, which contribute to tissue dysfunction, neuroinflammation, and tumor progression. Senolytic agents selectively target senescent cells by inhibiting SCAP-associated survival pathways, whereas senomorphic agents suppress SASP production without necessarily inducing cell death. Together, these therapeutic strategies aim to reduce chronic inflammation and mitigate aging-related pathological processes that may contribute to conditions such as cancer and neurodegenerative diseases. Created in BioRender. Santiago, M. (2025) https://BioRender.com/homive4.

In contrast, senomorphic agents modulate the phenotype of senescent cells rather than inducing their death (Vandenberk et al., 2011). Senolytics, which act through selective clearance of senescent cells, senomorphic compounds function by reducing or suppressing SASP factor secretion, thereby attenuating chronic low-grade inflammation and mitigating the deleterious effects of the senescent microenvironment (Kirkland and Tchkonia, 2020). Mechanistically, these agents target key signaling pathways, such as NF-κB, mTOR, JAK/STAT, and p38 MAPK, which regulate transcriptional activity responsible for SASP production and the maintenance of the senescent phenotype (Saliev and Singh, 2025; Kirkland and Tchkonia, 2017). Several natural compounds, including quercetin, fisetin, resveratrol, piperlongumine and curcumin, have demonstrated senoprotective activity by downregulating these pathways, highlighting their therapeutic potential in controlling age-related inflammation. Consequently, senomorphic agents represent a complementary strategy to senolytics, with the potential to modulate inflammaging and age-associated pathologies without directly inducing cell death (Suda et al., 2024). Figure 2 provides a schematic overview of the proposed therapeutic strategies and mechanisms of action of senolytic and senomorphic agents in senescent cells, emphasizing their reported effects on SCAP inhibition and modulation of the SASP. These concepts provide the foundation for classifying senotherapeutics into two main categories: natural and synthetic senolytic and senomorphic agents, each acting through distinct, although often overlapping, molecular mechanisms that contribute to aging and disease modulation.

### Natural senolytic and senomorphic agents

**FIGURE 3:**
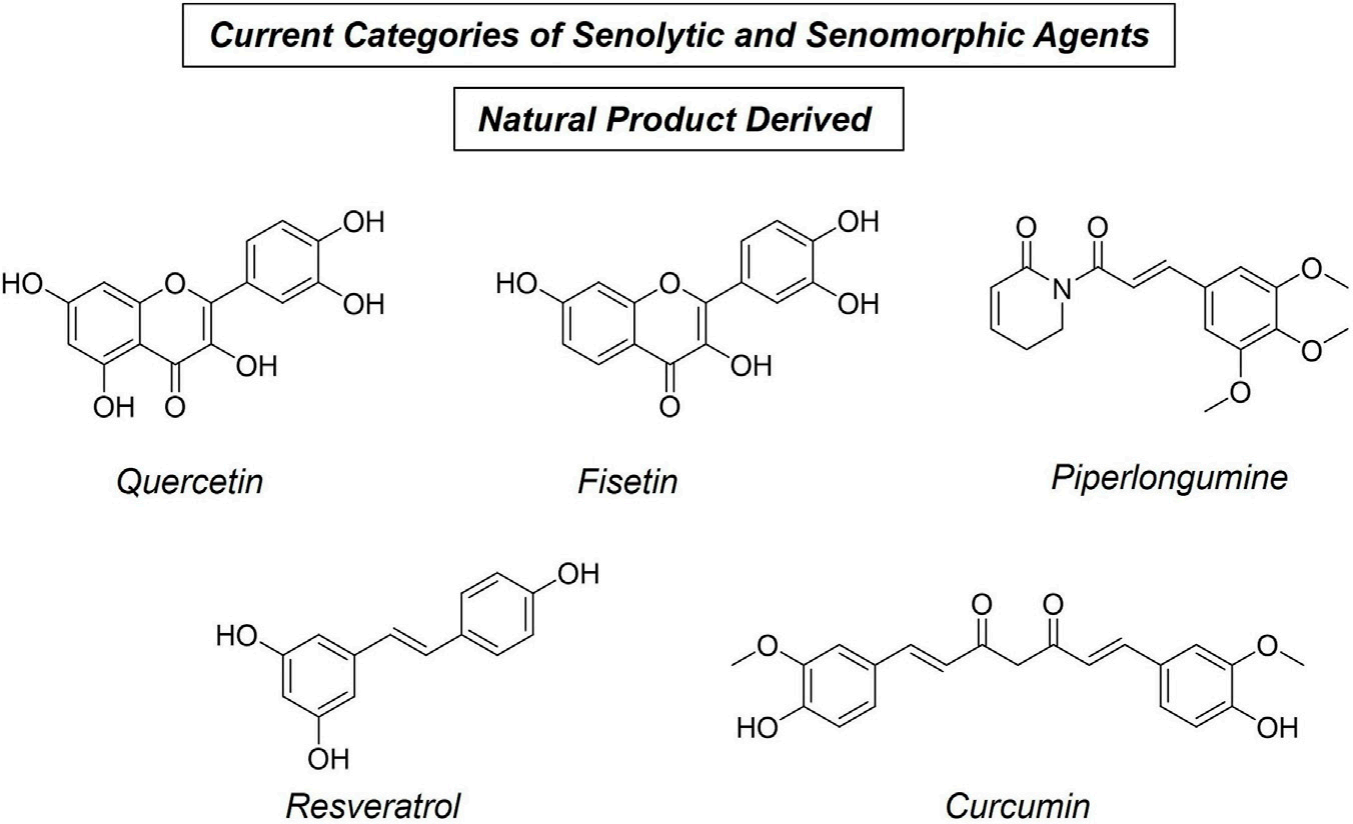
Classification of current senolytic and senomorphic agents derived from natural products. The figure illustrates representative molecular structures of selected natural agents, including quercetin, fisetin, resveratrol, curcumin, and piperlongumine, according to their reported senolytic or senomorphic activities.

Natural products (Table 4) have emerged as a rich source of senolytic agents, offering structural diversity and multi-target activity that makes them promising candidates for interventions related to aging. Several classes of natural compounds including polyphenols, flavonoids, terpenoids, and alkaloid derivatives, have been reported to exert senolytic or senomorphic effects by either promoting the selective elimination of senescent cells or modulating SASP-associated inflammatory signaling. Representative examples include quercetin, fisetin, piperlongumine, resveratrol, and curcumin (Figure 3), which have been identified as possessing potential senolytic and senomorphic activities. (Nehlin, 2023; Dong et al., 2024).

**TABLE 4:** Natural compounds with senolytic potential and their mechanistic targets in age-related diseases.

Natural product	Sources	Mechanisms of action	Classification and properties	Disease contexts	References
Quercetin	Apples, onions, berries	Inhibits BCL-2/BCL-xL family; reduces SASP factors and active SIRT-1	Senolytic phenolic compound of the flavonoid class. Antioxidant, anti-inflammatory and anti-carcinogenic.	Cancer, inflammation, neurodegeneration, and cell clearance. Regulate cellular senescence and aging related.	Aghababaei and Hadidi (2023), Deledda et al. (2022), Cui et al. (2022), Granado-Serrano et al. (2012), Alharbi et al. (2025), Zhou et al. (2023)
Fisetin	Strawberries, cucumbers, apples	Inhibiting pro-survival SCAP pathways, such as BCL-2/BCL-xL and PI3K/AKT/mTOR	Senolytic phenolic compound of the flavonoid class. Antioxidant, anti-cancer, anti-inflammatory and anti-microbial.	Cancer, neurodegeneration, senescent cell clearance.	Yousefzadeh et al. (2018), Xiao et al. (2021), Xiao et al. (2021), Tavenier et al. (2024), Lee et al. (2024), Zhu et al. (2017)
Piperlongumine	*Piper longum* (long pepper)	Increases ROS beyond the tolerance of senescent cells, apoptosis induction	Anti-inflammatory, anti-cancer, anti-neurodegenerative, and anti-senescent.	Senolytic alkaloids are commonly derived. Cancer, neurodegeneration (Parkinson), senescent cell clearance.	Rodrigues et al. (2021), Liu et al. (2018)
Resveratrol	Grapes, red wine, peanuts	Activates SIRT1/AMPK; modulates NF-κB and SASP (senomorphic more than senolytic)	Antioxidants, anti-inflammatory, anti-cancer, and anti-aging.	Phelonic compound of the stilbenoid class. Inflammation, neurodegeneration, cancer.	Galiniak et al. (2019), Panche et al. (2016)
Curcumin	*Curcuma longa* (turmeric)	Inhibits NF-κB, reduces SASP, antioxidant/anti-inflammatory (mainly senomorphic)	Antioxidants, anti-inflammatory, anti-cancer, and anti-aging.	Senolytic phenolic compound of the polyphenol class. Inflammation, cancer, neurodegeneration.	Aggarwal and Sung (2009), Panche et al. (2016), Huang et al. (2010), Li et al. (2019)

#### Flavonoids

Flavonoids are a large group of secondary metabolites found in fruits, vegetables, herbs, and other plant sources, characterized by a C6–C3–C6 skeleton consisting of two aromatic rings linked by a three-carbon bridge, which often forms a heterocyclic ring. Previous studies have shown that flavonoids exhibit antioxidant, anti-inflammatory, antiproliferative, and enzyme-modulating properties (Aghababaei and Hadidi, 2023). Within this category, the natural senolytic agents’ quercetin and fisetin have been identified. Both belong to the flavonoid class, a large group of secondary metabolites found in fruits, vegetables, herbs, and other plant-based sources.

#### Quercetin

Quercentin is a flavonoid abundant in apples, onions, grapes, berries, and green tea, whereas fisetin is enriched in strawberries, apples, persimmons, grapes, and cucumbers (Lagoumtzi and Chondrogianni, 2021). Several studies have suggest that this senolytic agent targets multiple pathways that confer senescent cells’ resistance to apoptosis. One proposed mechanism involves modulation of the anti-apoptotic BCL-2 family protein, including BCL-2/BCL-xL, thereby inducing selective apoptosis of senescent cells while preserving normal cells in either a proliferative or quiescent state (Miller et al., 2023). According to Cui et al. (2022), quercetin may exert protective effects in aging-related disease models through activation of SIRT1, a NAD^+^-dependent deacetylase that regulates multiple signaling pathways involved in the aging process, including NF-κB, PI3K/AKT, PGC-1α, and NLRP3. By enhancing SIRT1 activity, quercetin reduces oxidative stress, suppresses inflammation, improves mitochondrial function, and promotes autophagy, thereby exerting both senolytic and protective effects against cellular damage associated with aging (Richardson and Richardson, 2024).

#### Fisetin

Fisetin is a naturally occurring flavonoid found in various fruits and vegetables that has gained significant attention for its dual role as a senolytic and anti-cancer agent. Yousefzadeh et al. (2018) reported that fisetin selectively reduces senescent cell burden, potentially through modulation of SCAP-related pathways, including BCL-2/BCL-xL and PI3K/AKT/mTOR signaling (Deledda et al., 2022). Furthermore, Xiao et al. (2021) reported that fisetin suppresses the PI3K/AKT/mTOR signaling pathway, thereby inhibiting the proliferation, migration, and invasion of pancreatic cancer cells and reinforcing its therapeutic potential against both aging and cancer (Aghababaei and Hadidi, 2023; Calis et al., 2020). Beyond flavonoids, other polyphenolic natural products, including resveratrol and curcumin, have been investigated mainly for their senomorphic effects through modulation of inflammatory and stress-response pathways. In the comprehensive review “Fisetin as a senotherapeutic agent: Evidence and perspectives for age-related diseases,” an in-depth discussion of the pharmacological properties, mechanisms of action, and emerging clinical applications of fisetin as a potential senotherapeutic compound (Deledda et al., 2022).

#### Non-flavonoid polyphenols

Non-flavonoid polyphenols are plant-derived secondary metabolites isolated from various plants and are widely recognized for their antioxidant and anti-inflammatory properties. Their chemical structure is characterized by the presence of one or more aromatic rings bearing a hydroxyl (–OH) group. Among them, resveratrol and curcumin are prominent representatives with reported anti-inflammatory, anticancer, and anti-aging effects. Both exhibit multiple biological properties, including antioxidant, anti-inflammatory, anticancer, and anti-aging effects. They have been described primarily as senomorphic agents because they can regulate SASP expression and modulate signaling pathways such as NF-κB, mTOR, and SIRT1, thereby helping reduce chronic inflammation and cellular damage associated with aging (Valieva et al., 2022; Cui et al., 2022; Yousefzadeh et al., 2018).

#### Alkaloids

Piperlongumine is a natural alkaloid amide originally isolated from long peppers and has demonstrated biological activity in cancer and senescence models. It has been reported to selectively induce apoptosis in senescent cells by disrupting cellular redox balance and increasing oxidative stress, a mechanism also relevant to its anticancer activity. Recent studies have demonstrated the remarkable senolytic potential of piperlongumine and its structural analogs. These derivatives of the natural alkaloid display enhanced selectivity and potency toward senescent cells while retaining the characteristic α,β-unsaturated carbonyl scaffold responsible for their electrophilic reactivity and selective induction of apoptosis (Xiao et al., 2021). The development of piperlongumine analogs illustrates how structural modification of natural products can support the rational design of senolytic and senomorphic agents with improved selectivity and potency.

Likewise, combining natural and synthetic compounds represents a strategy to enhance senolytic efficacy, although further preclinical and clinical studies are needed to determine safety, efficacy, and translational potential. Although natural compounds such as quercetin, fisetin, piperlongumine, resveratrol, and curcumin have shown senolytic or senomorphic potential in preclinical models, their translation to human populations remains limited. Early human studies have evaluated senolytic approaches such as dasatinib plus quercetin and fisetin in selected clinical contexts, including Alzheimer’s disease risk, cognitive or mobility impairment and frailty in adult survivors of childhood cancer; however, these studies have not specifically addressed Caribbean Hispanic populations (Gonzales et al., 2023; Millar et al., 2025; Verdoorn et al., 2021; Hickson et al., 2019). In addition, clinical studies of natural polyphenols such as resveratrol and curcumin have explored cognitive and inflammatory outcomes, but these studies were not designed to directly test senolytic or senomorphic mechanisms. This gap is relevant given the accelerated demographic aging and high burden of age-related diseases, including dementia, reported in Caribbean Hispanic communities. Future studies should examine the safety, efficacy, bioavailability, and population-specific responses to these agents in this understudied population.

### Synthetic senolytic agents

Synthetic senolytic agents represent a growing class of rationally designed molecules developed to eliminate senescent cells by targeting key anti-apoptotic and pro-survival signaling pathways. These agents are designed to reduce senescent cell burden by promoting apoptosis in senescent cells. (Ge et al., 2021). Compared with many natural compounds, synthetic senolytics are often designed to exhibit greater potency and pathway selectivity by targeting well-defined molecular sites, including the BCL-2/BCL-xL family, PI3K/AKT signaling, and tyrosine kinase–mediated survival pathways.

One of the most studied examples is dasatinib,an FDA-approved tyrosine kinase inhibitor used in leukemia treatment that has been repurposed as senolytic agent. When combined with quercetin, a natural flavonoid, dasatinib acts synergistically to induce apoptosis in senescent cells. In preclinical models, the dasatinib and quercetin combination has been reported to reduce senescent cell burden across multiple tissues and improve physiological function. Early translational studies in humans and non-human primates have also reported reductions in selected markers of senescence and inflammation, although clinical efficacy and long-term safety remain under investigation (Lee et al., 2024; Zhu et al., 2015). Liu et al. (2018) reported that treatment with dasatinib and quercetin attenuates adipose tissue inflammation and improves metabolic function in aged mice, further highlighting the therapeutic relevance of senolytic interventions for age-related metabolic dysfunction. Another synthetic senolytic agent is navitoclax (ABT-263), a BH3 mimetic that inhibits the BCL-2/BCL-xL protein family and was originally developed as an anticancer compound. Navitoclax selectively promotes apoptosis in senescent cells by inhibiting BCL-2 family proteins and has demonstrated therapeutic potential in preclinical models, including the reversal of whole brain irradiation-induced blood–brain barrier disruption in mice (Gulej et al., 2023). However, safety findings from a phase 2a study in patients with relapsed or refractory lymphoid malignancies showed that navitoclax treatment was associated with hematologic toxicities, including thrombocytopenia (de Vos et al., 2021). Consistently, the REFINE phase 2 study evaluating navitoclax plus ruxolitinib in Janus kinase inhibitor-naïve patients with myelofibrosis further identified thrombocytopenia as a clinically relevant adverse event (Passamonti et al., 2026). These findings suggest that, although navitoclax has senolytic potential, dose optimization, treatment schedule, patient selection, and hematologic monitoring should be carefully considered before translating navitoclax-based senolytic strategies to aging populations.

Proteolysis-targeting chimeras (PROTACs), including BCL-xL-targeting navitoclax analogs, have emerged as an approach to improve senolytic selectivity by promoting targeted degradation of BCL-xL. This strategy may reduce platelet toxicity associated with conventional BCL-xL inhibitors, although further validation is needed (Jia et al., 2023). In a murine model of brain irradiation–induced dysfunction, Gulej et al. (2023) reported that navitoclax reduced senescence-associated alterations and improved selected functional outcomes, suggesting potential relevance for neurodegeneration-related mechanisms. Another class of synthetic compounds includes peptide-based approaches such as FOXO4-DRI, a synthetic peptide designed to disrupt the FOXO4–p53 interaction, thereby promoting apoptosis in senescent cells (Zhang et al., 2020; Huang et al., 2021). More recently, UBX compounds, including BTK-PROTAC degraders, have been developed to inhibit B-cell receptor signaling by targeting Bruton’s tyrosine kinase (BTK), representing a new frontier in senolytic drug design.

**FIGURE 4:**
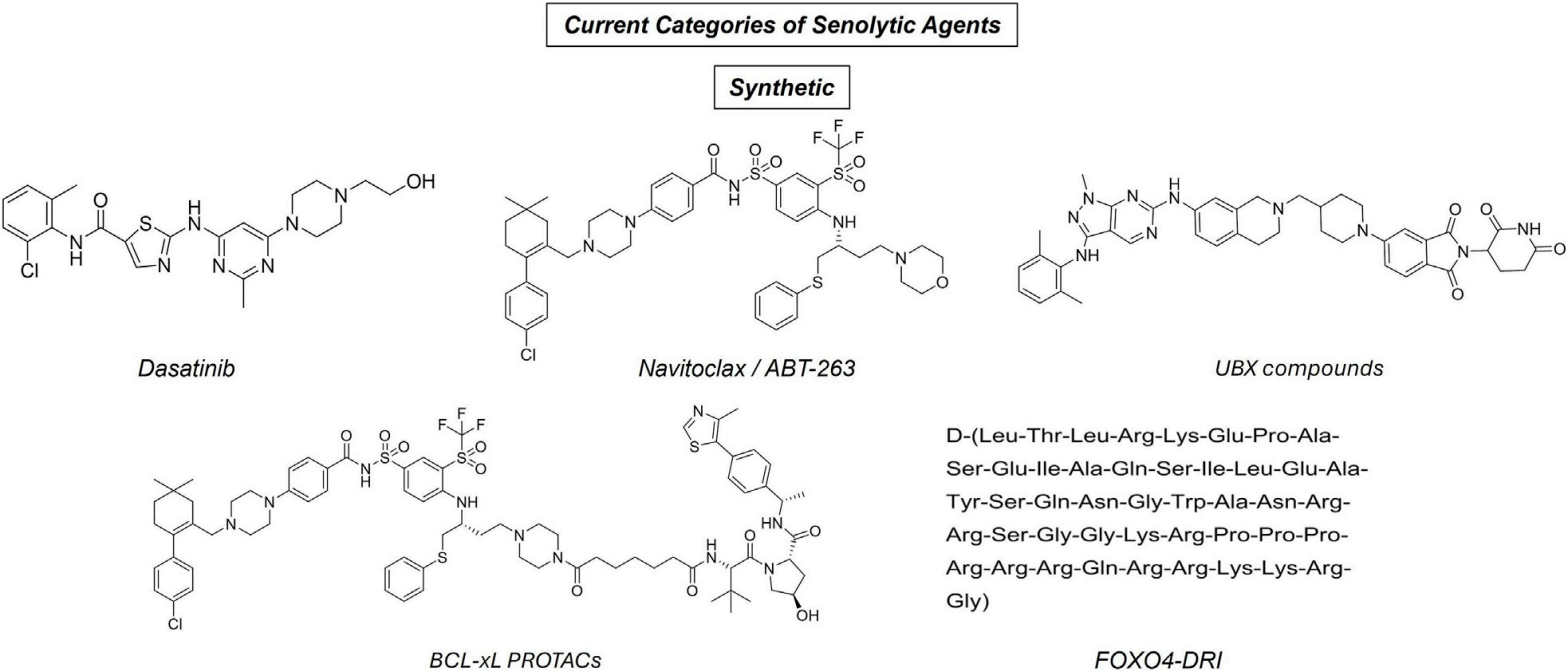
Current categories of synthetic senolytic compounds. Representative molecular structures of selected synthetic agents are shown according to their reported senolytic mechanisms of action.

These synthetic agents, illustrated in Figure 4, have shown potential for targeting cellular senescence and senescence-associated inflammation in preclinical models of aging, cancer, metabolic dysfunction, and neurodegeneration-related pathology. However, challenges remain in optimizing drug selectivity, reducing toxicity, and advancing them from preclinical studies to safe and effective clinical therapies for neurodegenerative and age-related diseases. Lorenzo et al. (2023) emphasized that the short- and long-term immunological consequences of senolytic treatment remain incompletely understood, particularly because senescent cells may influence immune regulation, responses to infection, and immunological memory. Although these advances are promising, challenges remain in optimizing drug selectivity, reducing toxicity, and advancing synthetic senolytics from preclinical studies to safe and effective clinical applications.

### Promising therapies tested in Caribbean Hispanic populations

Although Caribbean Hispanics remain substantially underrepresented in large-scale therapeutic trials for Alzheimer’s disease (AD), recent studies conducted within Caribbean Hispanic populations have begun to identify promising therapeutic approaches targeting neuroinflammation, neurodegeneration, and gut–brain axis dysfunction (Sepúlveda-Rivera et al., 2025; Sosa et al., 2023; Cox et al., 2026). While much of the available evidence remains preclinical, these investigations provide valuable insights into precision medicine strategies tailored to this high-risk and historically understudied population.

One notable example is the development of NeuroEPO Plus, a recombinant sialo-glycoprotein evaluated in Cuban patients with AD. NeuroEPO Plus is administered intranasally, thereby bypassing hepatic first-pass metabolism and gastrointestinal degradation while facilitating central nervous system delivery. Preclinical studies demonstrated that NeuroEPO reduced amyloid plaque burden, attenuated neuroinflammation, suppressed the expression of pro-inflammatory cytokines such as TNF-α and IL-1β, decreased oxidative stress, and limited neuronal apoptosis in transgenic AD mouse models (Sosa et al., 2023). Building upon these findings, Sosa et al. (2023) conducted a clinical study in patients with mild-to-moderate AD and reported improvements in cognitive performance following 48 weeks of treatment, without serious adverse events. Based on these encouraging results, NeuroEPO Plus received accelerated regulatory approval in Cuba. Ongoing and future studies are expected to compare NeuroEPO Plus with standard therapies such as donepezil and evaluate potential combination approaches (Sosa et al., 2023).

Beyond direct pharmacological interventions, growing evidence highlights the microbiota–gut–brain axis as a promising therapeutic target for age-related cognitive decline. Ge et al. (2021) demonstrated that age-associated alterations in the intestinal microbiome contribute to impaired gut–brain communication, reduced hippocampal neuronal activation, and memory dysfunction. Their work identified microbiome-derived medium-chain fatty acids as key mediators of inflammation through activation of the GPR84 receptor on peripheral immune cells, resulting in disrupted vagal signaling and impaired cognitive function. Importantly, the study identified Parabacteroides goldsteinii and microbiome-associated inflammatory pathways as potential therapeutic targets, supporting the development of microbiome-modulating interventions aimed at preserving cognitive health during aging. The authors further emphasized the role of chronic low-grade inflammation, peripheral macrophage activation, dietary influences on microbiome composition, and the potential neuroprotective effects of incretin-based therapies.

Recent advances have also highlighted the intersection between gut microbiota, epigenetic regulation, and chronic inflammation. Emerging evidence suggests a bidirectional relationship between microbiome composition and non-coding RNA (ncRNA)-mediated regulatory pathways, particularly microRNAs (miRNAs), which influence inflammatory responses and disease progression (Zhang et al., 2025). Although these mechanisms have been studied primarily in inflammatory bowel disease and colorectal cancer, their relevance to AD is increasingly recognized, given the central role of neuroinflammation in disease pathogenesis (Zhang et al., 2025). Supporting this concept, recent studies in Puerto Rican older adults with AD identified significant gut microbiota dysbiosis associated with greater cognitive impairment severity (Zhu et al., 2017). These findings suggest that microbiome alterations may contribute to disease progression and highlight the potential utility of miRNAs as both biomarkers and therapeutic targets within the microbiota–gut–brain axis (Lin et al., 2025). Consequently, ncRNA-based approaches may offer novel opportunities to improve biomarker discovery, patient stratification, and precision therapeutic development in Caribbean Hispanic populations affected by AD.

Collectively, these emerging therapeutic strategies underscore the growing potential of targeting neuroinflammation, microbiome dysbiosis, and epigenetic regulatory mechanisms to mitigate cognitive decline and AD progression in Caribbean Hispanic populations. While current evidence remains limited and largely exploratory, findings from studies involving NeuroEPO Plus, microbiome-modulating interventions, and ncRNA-based approaches provide a foundation for the development of precision medicine strategies tailored to the unique biological and demographic characteristics of this population. Future large-scale clinical and translational studies are needed to validate these approaches, determine their long-term efficacy, and establish their role in reducing the disproportionate burden of AD among Caribbean Hispanics.

## Concluding remarks

It is becoming increasingly evident that weathering—the cumulative physiological wear and tear resulting from chronic exposure to social, environmental, and psychological stressors—has measurable biological consequences. These effects manifest through intertwined biological and social mechanisms that jointly shape individual and population health trajectories. Members of racially and ethnically disadvantaged or underserved minority groups often experience sustained psychosocial stress stemming from systemic prejudice, discrimination, and socioeconomic adversity. Over time, this chronic stress burden contributes to epigenetic modifications that alter gene expression, thereby influencing biological function and promoting processes associated with premature aging, chronic low-grade inflammation, and increased susceptibility to chronic diseases.

The biological pathway linking weathering to adverse health outcomes is mediated by several interconnected processes, including activation of biological stress responses (e.g., dysregulation of the hypothalamic-pituitary-adrenal axis), epigenetic remodeling, and persistent inflammatory signaling. These mechanisms collectively accelerate cellular senescence and tissue dysfunction; hallmark features of aging-related decline. However, the biological effects of weathering cannot be fully understood without considering the powerful influence of social determinants of health. Structural inequities—such as disparities in healthcare access, quality of care, educational opportunities, and economic stability—exacerbate the biological toll of chronic stress, particularly in underserved and ethnically diverse populations like Caribbean Hispanics.

Ethnic and racial bias in the distribution and quality of health services further magnifies these disparities. Individuals from minority and low-income communities often face limited access to preventive care, underdiagnosis, or suboptimal treatment, leading to poorer health outcomes and accelerated biological aging. Beyond the healthcare system, factors such as neighborhood conditions, built environment, and lifestyle determinants—including access to nutritious food, clean water, and opportunities for physical activity—shape the trajectory of weathering and its long-term health effects.

Weathering represents a complex cycle of reciprocal interactions between biological vulnerability and social adversity. The feedback loops between chronic stress exposure, inflammation, and accelerated aging reinforce health inequities across generations. Understanding this bidirectional relationship is essential for developing precision medicine strategies that account for both molecular and social dimensions of health—particularly within Caribbean Hispanic populations, where addressing these intersecting determinants may hold the key to mitigating premature aging and promoting health equity across the lifespan.
