# Supplementary material for: Accelerated biological aging: the role of chronic inflammation, weathering and novel therapeutic strategies
Source: Front Aging. 2026 Jul 21;7:1863781. doi: 10.3389/fragi.2026.1863781 (PMC13433523; doi:10.3389/fragi.2026.1863781)
Supplement: Supplementary file 1 [file DataSheet1.pdf]

Supplementary Material

1 Supplementary Material

Supplementary Material should be uploaded separately on submission. Please include any supplementary data, figures and/or tables.

Figure S1: Annual publication trends in accelerated biological aging, inflammaging, and senotherapeutic strategies. The figure shows the number of selected references published per year and provides a descriptive overview of the temporal distribution of the literature included in this narrative review.

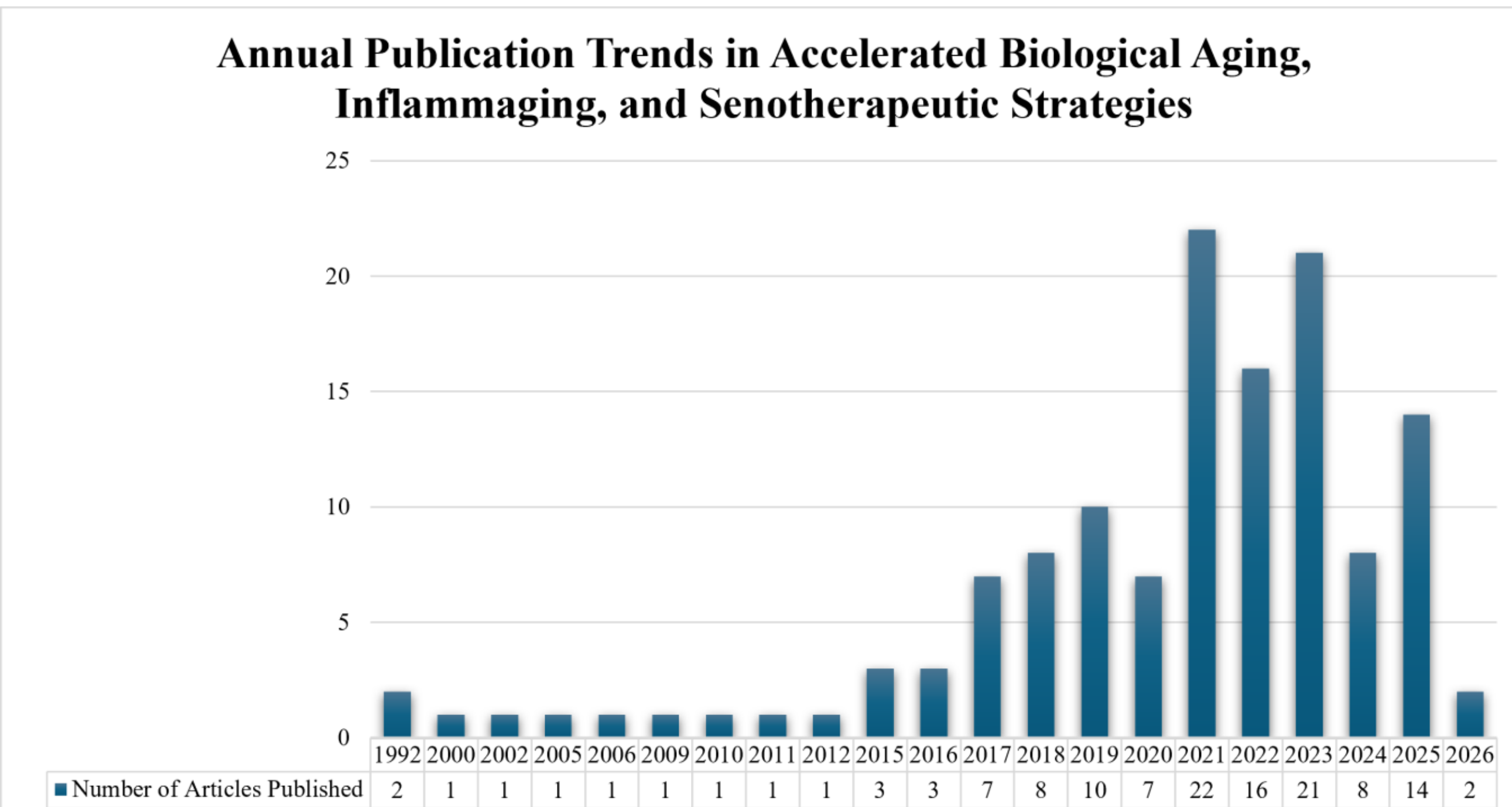

Table S1. FDA-approved siRNA therapeutics with their clinical indications, mechanisms of action, year of approval, and gene targets. The table is based on the FDA Drug index references.

Drug	FDA Approval date	Condition Approved	Mechanism of action	Gene silenced
Onpattro® (Patisiran)(49)	2018	Polyneuropathy of hereditary transthyretin-mediated	The siRNA binds transthyretin messenger RNA in liver cells, leading to cleavage by the RISC complex. This reduces	*TTR*

		amyloidosis in adults.	transthyretin protein production and lowers amyloid deposits in nerves and tissues	
Givlaari® (Givosiran)(50)	2019	Adults with acute hepatic porphyria (AHP).	The siRNA binds aminolevulinic acid synthase one messenger RNA in the liver. This decreases production of aminolevulinic acid and porphobilinogen, preventing buildup of neurotoxic intermediates	*ALAS1*
Oxlumo®<br><br>(Lumasiran)(51)	2020	Primary hyperoxaluria type 1 (PH1) to lower urinary oxalate levels in pediatric and adult patients.	The siRNA binds hydroxyacid oxidase one messenger RNA, reducing glycolate oxidase levels. This lowers oxalate production and prevents kidney stone formation and renal damage	*HAO1*
LEQVIO® (inclisiran)(52)	2021	Clinical atherosclerotic cardiovascular disease (ASCVD), who require additional lowering of low-density lipoprotein cholesterol (LDL-C).	The siRNA binds proprotein convertase subtilisin kexin type nine (PCSK9) messenger RNA. This reduces PCSK9 protein levels, increases LDL receptor recycling, and lowers LDL cholesterol	*PCSK9*
Amvuttra®<br><br>(Vutrisiran)(53)	2022	The polyneuropathy of hereditary transthyretin-mediated amyloidosis in adults, the cardiomyopathy of wild-type or hereditary transthyretin mediated	The siRNA binds transthyretin (TTR) messenger RNA after subcutaneous delivery. This decreases TTR protein levels and reduces amyloid accumulation	*TTR*

		amyloidosis in adults		
Rivfloza ® (nedosiran)(54)	2023	Approved for the treatment of primary hyperoxaluria type 1 (PH1) in adults and children ≥9 years of age with preserved kidney function (eGFR ≥30 mL/min/1.73 m²).	siRNA that selectively degrades LDHA mRNA in hepatocytes, lowering hepatic lactate dehydrogenase A levels and reducing oxalate production.	*LDHA*
Qfitlia®<br><br>(Fitusiran)(55)	2025	Hemophilia A/B (prophylaxis).	A siRNA that silences antithrombin (SERPINC1), restoring balance to blood clotting	*SERPINC1*
